# The Institutional Worker

**Published:** 1920-01-17

**Authors:** 


THE
### INSTITUTIONAL WORKER
**Being a Special Supplement to "The Hospital"**

### OUR BUREAU OF INFORMATION.

**Rules for Correspondents.**

1. Every letter must be accompanied by the coupon to be cut from the back cover (inside page) of The Hospital, current issue, and must contain the name and address of the correspondent with pseudonym for publication if desired. Replies by post cannot be given save under exceptional circumstances at the Editor's discretion.
2. Letters from Approved Homes in reply to special needs published in the Bureau should state terms and full particulars, and be sent prepaid under cover to the Editor of the Bureau with name written across coupon for identification.
3. Proprietors of Homes which have not yet been entered on the List of Approved Homes, but have spare accommodation likely to suit special needs, are invited to write for an application form for registration. The fee for registration, which includes two announcements of the Home in the Bureau and other privileges, is 10s.
4. All communications to be addressed to the Editor of The Hospital, 28 Southampton Street, Strand. London, W.C. 2, and marked "Bureau of Information."

### INSTITUTION FACTS AND FIGURES.

### WHAT IS WRONG WITH OUR HOSPITALS?

**A MISSING LINK.**

By B. W.

Considerable anxiety is apparently felt by many who are responsible for the administration of our hospitals throughout the country, because the incomes of the hospitals have not expanded in proportion to the abnormal increase in the cost of all requisites for their maintenance. Here and there the voices of some of the most ardent supporters of the voluntary system may be heard speaking in favour of State support and State control as the only solution of the problem.

If the system be sound, and if it reflects the wish of the people of this country, then surely it should not perish from starvation. Success in any commercial enterprise results from satisfying conditions, which may be crystallized under two main heads:—(1) Systematic advertisement. (2) Ability to supply the popular need.

As regards the former, it is evident that as a whole the hospitals are fairly well advertised, but can it be truly said that the latter condition is fully met?

As the State made provision for dealing with those diseases which are obviously a public danger, overlooking serious illnesses and injuries, the neglect of which apparently suggested no danger to the State, so also voluntary effort has provided hospital accommodation of a general character for one section only of the community, the one most obviously in need of it. "Free to the deserving poor" has been the invariable cry; free to the poor not otherwise provided for. The varying degrees of poverty have been almost forgotten, for the provision made for those who can pay for part or the whole of their maintenance, or those who can pay for their maintenance and something toward medical attendance is small indeed. The years of war have done much to emphasize this omission, for during this period many changes have taken place in the fortunes of the community. The spirit which underlies the maintenance of hospitals by voluntary effort is the spirit of the brotherhood of sickness, of which, sooner or later, everyone becomes a member. The secret of the regeneration of the financial status of the hospitals seems to lie in the recognition of this fact and in adopting a course which will meet the need arising from altered circumstances.

To-day there are a great many more people than there used to be who can pay something for their maintenance in hospital, and there are numbers who would be glad to have hospital accommodation if only they were permitted to pay. At Hampstead a section of the hospital is reserved for those who can pay from two to four guineas a week; the patients are attended by their own doctors, though the poorer of them not infrequently have the services of consulting physicians and surgeons without fee. Similar arrangements and modifications of them are made at other hospitals, but such accommodation is hopelessly inadequate.

Without disturbing in any way the relations which exist between the honorary visiting staffs and the hospitals, and following and extending a plan which has survived the test of experience, provision might be made for (1) free maintenance, nursing, and medical attendance at the discretion of the almoner, (2) a nominal charge for maintenance and nursing and free medical attendance, (3) full charge for maintenance and nursing and if necessary free medical attendance, (4) charges to suit the means of patients and in accordance with character of accommodation, without medical attendance, in which case the wards or rooms would be free to the medical practice of the district.

The plan of making a nominal charge for maintenance has long been recognised in many hospitals, in some the contribution takes the form of certain foods; the practice, too, of charging out-patients for medicine is becoming more general.

In effect the patients who would be asked to pay toward the cost of their maintenance would be those in similar circumstances to the patients who at present are asked to pay nothing and are freely treated by the honorary staff. Free maintenance for these cases is unnecessary; it is the skilled medical attendance and the services of the surgeon which cannot be paid for by this section of the poor.

The modification in the manner outlined of the existing system would do much toward consolidating the efforts of the medical practitioner and the hospitals to combat disease, and would form the basis of an appeal to the public so strong that all financial difficulties would be swept away, and in time the hospitals might become largely self-supporting. All the advantages of individual effort would be retained, and the disadvantages of a State department averted.

---

**A Helpful Crutch.**

One of the most comfortable and at the same time a scientifically constructed crutch is that designed by Colonel F. Weldon, of Reading. The advantage which this crutch has over many other types is that the head has been constructed on rational lines; it conforms to the oblique direction of the axilla, while the bases of the crutches are parallel to each other and to the line of progression. All risk of crutch paralysis is thus eliminated. The handles of the crutches being also kept parallel to each other, the arms are maintained in a position best calculated to ensure continuous exercise with minimum fatigue. Each crutch is provided with two legs and a curved segmental base so proportioned and adjusted that the body is mainly propelled by gravitation. These crutches can be fitted with elbow-rests and seats if required. They may be obtained of Messrs. Cory Bros., Mortimer Street, W. 1.—Tony.

### Question for January.

### UTILISING VOLUNTARY HELP

How would you deal with offers of voluntary part-time help in connection with your hospital from professional men, clerks, &c.? Give suggestions how their assistance might be utilised.

**(A minimum payment of 10s. 6d. will be made for each published answer.)**

RULES.

The following rules must be observed:—

1. Contributions must be written on one side of the paper. Conciseness and terseness are desirable features. The MS. must bear the name and address of the sender and be accompanied by coupon to be cut from the back cover (inside page) of the current issue of THE HOSPITAL. A pseudonym must be chosen if the name is not to be published.

2. Contributions must be addressed to the Editor of THE HOSPITAL Institutional Worker Supplement, 28 & 29 Southampton Street, Strand, London, W.C. 2, should reach him before the end of the current month, and be marked in the left-hand corner "Facts and Figures."

### QUESTIONS INVITED.

IN connection with our Question Box, we offer every month five shillings each for the best questions which are sent in for consideration. The questions may be on any institutional subject and concern any department, from the secretary's to the porter's, or the matron's to the domestic staff; the one essential is that they must be practical and deal with points that have a definite relation to hospital or institutional administration, upkeep, finance, or management. Points relating to artificers' work, housekeeping, the laundry, out-patients, and other practical matters will be welcome. It is hoped that thus, when difficult or doubtful points arise in the course of their work, institutional workers will be encouraged to put them in the form of a question and send them to the Editor, THE HOSPITAL Bureau of Information, 28 & 29 Southampton Street, Strand, London, W.C. 2, marked "Question Box."

The best questions will be published in due course and our readers will be given the opportunity of answering them. Thus all institutional workers may be able to cooperate with the view of helping the work and smoothing the difficulties of each other. In short, we want the Question Box of the *Institutional Worker* to be freely used by every worker habitually.

### ENQUIRIES AND ANSWERS.

### *THE SICK AND IN NEED.*

### Uncared-for Child.

WE suggest that you apply to the Secretary of the National Children Adoption Association on behalf of the orphaned child in whom you are interested. We understand there is no age limit for prospective inmates, and no distinction of class, but every endeavour is made to place the children in the same walk of life as that in which they were born. The offices of the Association are at 19 Sloane Street, London, S.W. 1. The Hostel is at Tower Cressy, Campden Hill.—"A WORKER."

### Permanent Home for Nurse.

APPLY for admission to King Edward VII. Memorial Home, Clapham Park and Norland Square. Address your application to the Secretary, at 15 Buckingham Street, Strand, W.C.—"DISABLED."

### Resident Nurses' Club.

A NURSES' CLUB in the district you require is the Kensington Gardens Nurses' Club, 56 and 57 Kensington Gardens Square, W. 2. It is open to resident and non-resident members. Apply to the Principal.—NURSE A.

### *MISCELLANEOUS.*

### An Up-to-date Bed Warmer.

AN electric bed-warmer which has stood the test of two years' practical use is produced by Electric Fabrics, 125 to 128 London Road, Southwark, London, S.E. 1. The warmer is 60 inches long and 30 inches wide, and is therefore large enough to warm at once the whole area upon which the patient reclines—a unique advantage. The current is supplied from a lamp socket at the head of the patient's bed, and the warmer can be supplied to suit the voltage available. The heating elements are enclosed in a fire-resisting fabric, and the whole is covered with a washable jacket. The price is £5 5s.—"COMFORT."

### Address Wanted.

THE address of the Royal Institute of Public Health is 37 Russell Square, London, W.C. 1. Write to the Secretary of the Institute, who will give you all the information you require.—G. E.

### Laundry Plant.

THERE are so many makers of laundry machinery that it is quite impossible for us to give you a complete list. Messrs. Manlove, Alliott and Co., of Nottingham, are a well-known firm, and their work has great merit. Messrs. A. L. Marshall, of Carlton, near Nottingham, specialise in some admirable automatic ironing machines and washers. They have for sale just now a quantity of rebuilt laundry machinery, inspection of which might be instructive.—"ECONOMY."

### *EMPLOYMENT AND TRAINING.*

### Sister-Tutor Scholarships.

THERE is no need for you to have had a College education to become a sister-tutor. Scholarships are awarded by the College of Nursing, 7 Henrietta Street, Cavendish Square, W. 1. Write to the Secretary of the College for particulars of entry. You might also write to the Secretary of King's College for Women, Campden Hill, W.—PROGRESSIVE.

### Training-School in New Zealand.

YOU might apply for a vacancy at the Auckland Hospital, New Zealand. The hospital contains 340 beds, and provides a four years' training. No premium is required, and the salary commences at £10 for the first year, rising to £45 for the fourth year.—CHRISTCHURCH.

### Nurses' Missionary League.

THE address of the Nurses' Missionary League is Sloane Gardens House, 52 Lower Sloane Street, S.W. Yes; nurses in training who purpose becoming foreign missionaries are accepted as volunteer associates. Write to the General Secretary, Miss Richardson, who will advise you.—EAGER.

### Social Welfare.

AN appointment as health visitor or for child-welfare work would probably appeal to you. Your general training and Central Midwives Board certificate are a good qualification for the latter work. If you are interested you should write to the National Association for Child Welfare, 4 Tavistock Square, W.C. For health work the certificate of the Royal Sanitary Institute, Buckingham Palace Road, S.W., is probably the most valuable. Regulations for the training of health visitors are published by the Board of Education, and may be obtained from His Majesty's Stationery Office, Imperial House, Kingsway, W.C. 2. The price is 1½d. post free.—TRAINED NURSE.

### Midwifery Training.

IT will be necessary for you to take four months' training to qualify for the certificate of the Central Midwives Board. Apply to the City of London Maternity Hospital, City Road, E.C., and Fulham Midwifery School, 211 New King's Road, Fulham, S.W.—BASINGSTOKE.

### General Training with Massage and Electricity.

THE Essex County Hospital, Colchester, receives candidates for three years' training from the age of twenty-one to thirty-five. Two nurses per year are sent by arrangement for training in massage and electricity, returning to finish training and make up the six months at the end. Application should be made to the matron, by whom a personal interview is desired.—KATIE.

